# Posterior Leaflet Overlay Patch Reinforcement for Mitral Valve Posterior Tethering: The Elbow Patch Repair

**DOI:** 10.5761/atcs.nm.25-00068

**Published:** 2025-05-24

**Authors:** Hyeon A. Kim, Jae Suk Yoo

**Affiliations:** 1 Department of Thoracic and Cardiovascular Surgery, Ewha Womans University Seoul Hospital, Seoul, Republic of Korea; 2 Department of Thoracic and Cardiovascular Surgery, Asan Medical Center, University of Ulsan College of Medicine, Seoul, Republic of Korea

**Keywords:** mitral valve insufficiency, atrial fibrillation, mitral valve repair

## Abstract

The scarcity of leaflet tissue and restricted systolic motion remain challenges in mitral valve repair. In addition to functional or secondary mitral regurgitation, atrial functional mitral regurgitation, characterized by chronic atrial fibrillation, preserved left ventricular function, and atriogenic leaflet tethering, exacerbates leaflet scarcity, complicating mitral valve repair. To address this, we introduce the “elbow patch repair,” a novel technique using an autologous pericardium overlay patch to reinforce the posterior mitral valve leaflet. A 65-year-old male patient with chronic atrial fibrillation and severe mitral regurgitation consistent with atrial functional mitral regurgitation underwent the “elbow patch repair” combined with annuloplasty and neochordae placement. This approach effectively managed posterior mitral valve leaflet deficiency and restored the coaptation surface. The “elbow patch repair” offers a straightforward and effective solution for leaflet shortage in atrial functional mitral regurgitation and select cases of Carpentier Class IIIb. Further studies are needed to assess its long-term durability.

## Introduction

The paucity of leaflet tissue and systolic restricted leaflet motion often complicate mitral valve repair (MVr). In addition to the conditions categorized within Carpentier Class IIIb^1)^—ischemic myocardial disease, idiopathic dilated cardiomyopathy, and end-stage heart disease—atrial functional mitral regurgitation (AFMR) has become an increasingly recognized condition. It occurs when the geometry and function of the left ventricle (LV) are relatively preserved compared to other left heart diseases, particularly in the setting of chronic atrial fibrillation (AF). AFMR is characterized by significant left atrial (LA) remodeling and atriogenic leaflet tethering, resulting in ineffective MV coaptation.^2,3)^ These features make MVr challenging in both AFMR and Carpentier Class IIIb. The “elbow patch repair,” involving reinforcement of the posterior mitral valve leaflet (PMVL) with an autologous pericardium overlay patch, has been proposed as a potential approach to address tissue deficiencies and restore an adequate coaptation surface.

## Case Presentation

**Fig. 1:**
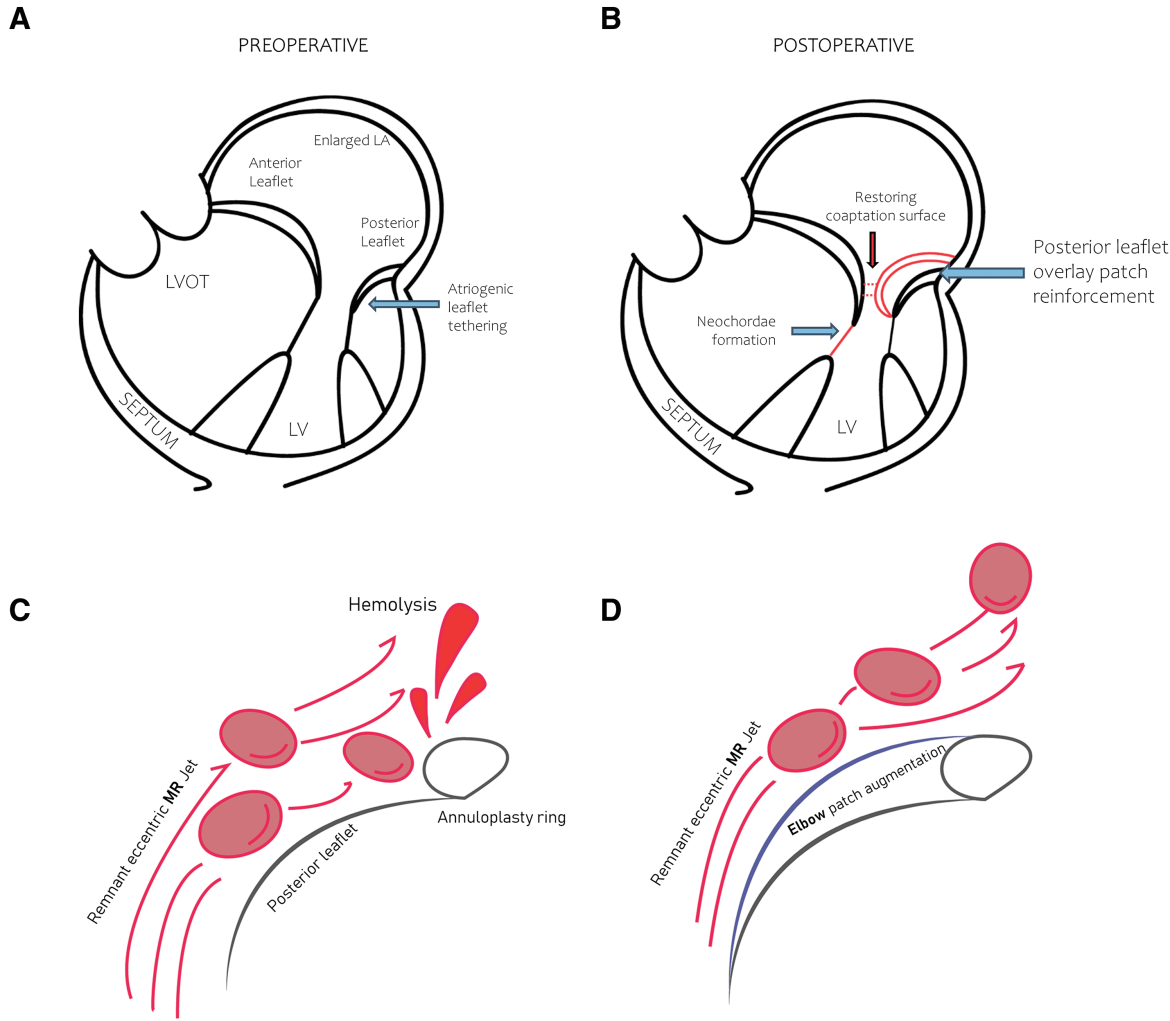
Schematic representation of the “elbow patch repair.” (**A** and **B**) Posterior leaflet overlay patch reinforcement using autologous pericardium offers a sufficient coaptation surface. (**C** and **D**) Reduces the risk of hemolysis by avoiding MR jet collision with the artificial ring. LA: left atrial; LV: left ventricle; LVOT: left ventricular outflow tract; MR: mitral regurgitation

**Fig. 2:**
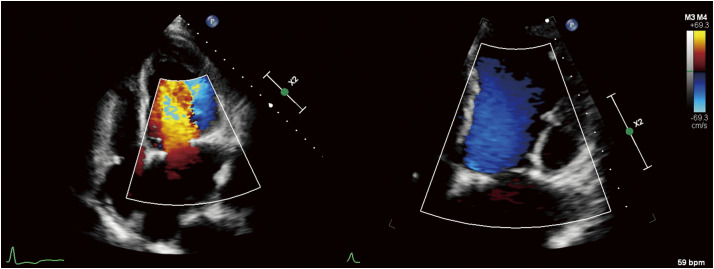
Postoperative echocardiography findings. Postoperative echocardiography revealed no residual MR, an MV area of 2.6 cm^2^, a transmitral pressure gradient of 10/2 mmHg, and an LV EF of 40%. EF: ejection fraction; LV: left ventricle; MR: mitral regurgitation; MV: mitral valve

A 65-year-old male patient presented with chronic persistent AF and severe MR. Initial evaluation with transthoracic echocardiography revealed a dilated LA and mitral annulus with preserved ejection fraction heart failure, which was likely associated with AFMR, marked by posterior leaflet tethering and prolapse of the anterior leaflet. An annuloplasty with a complete ring (CG Future Ring 34 mm; Medtronic, Minneapolis, MN, USA) was performed for annular remodeling, accompanied by the formation of 2 neochordae on the anterior leaflet to address the anterior prolapse. Despite these interventions, the restrictive PMVL tissue due to atriogenic tethering suggested that a sufficient coaptation area was not secured. To address the insufficiency, an “elbow patch repair” was performed, involving reinforcement of the posterior leaflet with an autologous pericardial overlay patch to secure the coaptation surface. The pericardial patch was tailored intraoperatively to fit the posterior leaflet geometry, typically measuring 20–30 mm in width and 10–15 mm in height. Continuous 5-0 polypropylene suture was placed to cover the posterior annuloplasty ring and approximately 2 mm from the free edge of the residual leaflet, targeting the rough zone (see **Supplementary Video** and **Fig. 1**). Care was taken to avoid excessive patch redundancy and to maintain an appropriate coaptation height, preventing leaflet dysfunction due to overaugmentation. Additionally, the patient underwent a concomitant biatrial maze procedure using cryoablation and resection of the LA appendage, as well as tricuspid valvuloplasty. Postoperative echocardiography showed no remnant MR, an MV area of 2.6 cm^2^, and a transmitral pressure gradient of 10/2 mmHg, alongside LV dysfunction with an ejection fraction (EF) of 40% (**Fig. 2**). Treatment for heart failure (HF) was optimized with optimal medical therapy, and the patient was subsequently discharged with follow-up arranged in the outpatient department.

## Discussion

Functional or secondary MR arises from left-sided heart dysfunction, resulting in an imbalance between leaflet tethering and closing forces, which leads to inadequate coaptation rather than intrinsic structural changes in the valve itself. Among these, AFMR represents a less common but increasingly recognized subset, occurring when the geometry and function of the LV are relatively preserved, often in the setting of chronic AF.

Progressive LA remodeling driven by chronic AF draws the mitral annulus toward the basal wall of the LV, resulting in atriogenic leaflet tethering and systolic restriction of leaflet motion.^2,3)^ These changes further reduce PMVL tissue, and MV replacement often becomes an inevitable option.

Carpentier’s textbook describes repair techniques for Class IIIb MR, noting that procedures such as leaflet extension, secondary chordae cutting, or papillary muscle repositioning,^1)^ also applicable to AFMR, can be utilized but often involve technical challenges. While pericardial patch extension of the PMVL is the most commonly utilized technique among these,^4)^ the elbow patch repair is technically less complex, reduces the risk of leaflet injury, does not require excision, and minimizes the need for precise anatomical tailoring.

Regarding resistance to calcification, we prefer to use fresh autologous pericardium, as several studies have discussed the pros and cons compared to glutaraldehyde-fixed patches.^5,6)^

The “elbow patch repair,” a posterior leaflet overlay patch reinforcement using autologous pericardium, provides a practical solution for PMVL tissue deficiency. This technique restores the coaptation area and redirects remnant MR flow from eccentric to concentric (**Fig. 1**). Moreover, by integrating autologous pericardium with an artificial ring, it effectively reduces the risk of hemolysis by preventing residual MR jet collision with the ring.^7)^

A conceptually similar approach had already been introduced by M. Tabata, known as the double-leaflet technique.^8)^ This approach offers a technically straightforward and more physiologic repair by anchoring the patch to the papillary muscle and preserving subvalvular continuity. However, as noted in their study, its feasibility may be limited when more than half of the posterior leaflet requires augmentation, due to the need for direct attachment to the papillary muscle. In contrast, the elbow patch technique attaches the patch to the rough zone of the leaflet, making it suitable for both partial and extensive posterior leaflet deficiencies.

## Conclusion

The “elbow patch repair” offers a technically simple and effective option for addressing PMVL deficiency in selected cases of AFMR and Carpentier Class IIIb MR. By reinforcing the posterior leaflet with autologous pericardium, this technique restores coaptation while minimizing technical complexity and potential complications. Nonetheless, the long-term durability of this repair technique remains to be thoroughly examined.

## Declarations

### Ethics approval and consent to participate

Application for waiver of consent for the publication of this case report was approved by the ASAN Research Information System Sciences Institutional Review Board (Protocol No.: S2024-0341-0001, approved February 21, 2024).

### Funding

No funding to declare.

### Data availability statement

The data that support the findings of this study are available from the corresponding author upon reasonable request.

### Authors’ contributions

H. A. Kim contributed to data curation, analysis, investigation, methodology, visualization, and manuscript drafting. J. S. Yoo, as the corresponding author, was responsible for supervision, conceptualization, project administration, validation, and manuscript review and editing. All authors reviewed and approved the final version of the manuscript and agreed to be accountable for all aspects of the work.

### Disclosure statement

The authors declare that they have no competing interests.

## Supplementary Materials

Supplementary video Surgical video demonstrating posterior leaflet overlay patch reinforcement using autologous pericardium.
